# Brief Clinical Report: Hypophosphatasia—Diagnostic Considerations and Treatment Outcomes in an Infant

**DOI:** 10.1155/2018/5719761

**Published:** 2018-04-01

**Authors:** Sara Duffus, Bradly Thrasher, Ali S. Calikoglu

**Affiliations:** ^1^Department of Pediatrics, University of North Carolina, Chapel Hill, NC, USA; ^2^Division of Pediatric Endocrinology, Children's Hospital at Erlanger, Chattanooga, TN, USA; ^3^Division of Pediatric Endocrinology, University of North Carolina, Chapel Hill, NC, USA

## Abstract

Hypophosphatasia (HPP) is a rare, inherited metabolic bone disorder characterized by low serum alkaline phosphatase activity and impaired bone mineralization. Clinical manifestations and severity of symptoms vary widely in HPP, ranging from in utero death to isolated dental manifestations in adults. Treatment with enzyme replacement therapy has been reported to improve outcomes in perinatal, infantile, and childhood forms of HPP. Here, we present a case of a boy with poor linear growth, mild limb bowing, and radiographic rickets who was diagnosed with HPP before 6 months of age. Treatment with enzyme replacement therapy was initiated at 7 months of age, after which significant improvements in radiographic findings and linear growth were demonstrated. This case highlights several important challenges in the diagnosis, classification, and management of HPP.

## 1. Introduction

Hypophosphatasia (HPP) is a rare, inherited metabolic bone disorder characterized by low serum alkaline phosphatase (ALP) activity and impaired bone mineralization [1]. HPP is caused by loss-of-function mutations within the gene that encodes the tissue-nonspecific alkaline phosphatase (TNSALP) [1–3]. Extracellular accumulation of TNSALP natural substrates occurs, leading to inhibited mineralization of both teeth and bone [3]. The incidence of HPP varies widely depending on the genetic mutations found within a given population but has been estimated to range from approximately 1 in 2,500 to 1 in 300,000 live births [4, 5].

Clinical manifestations and severity of symptoms vary widely in HPP, ranging from in utero or neonatal death to isolated dental manifestations in adults [1, 6]. The severity of symptoms is generally worse when the disease presents earlier in life and depends on the degree of skeletal complications present [1, 7]. Traditionally, patients have been classified into five principal forms of HPP based on the presence of skeletal disease and age at presentation: perinatal, infantile, childhood, adult, and odonto-HPP (Table 1) [6, 8]. Additionally, benign perinatal HPP (BP-HPP) has been described, in which skeletal disease is detected in utero or at birth, but there is a mild postnatal course with spontaneous improvement in symptoms [9]. Treatment with enzyme replacement therapy, modified human TNSALP protein, has been reported to improve outcomes in both life-threatening perinatal and infantile forms of HPP, in addition to older children with childhood HPP [10–12].

Here, we present a case of mild but progressive HPP, which was misdiagnosed as nutritional rickets at 2 months of age. HPP diagnosis was subsequently established by 6 months of age. In addition to describing the clinical, radiographic, and biochemical features of this case, we will also report the short-term outcomes of treatment with modified human TNSALP protein.

## 2. Case Presentation

The patient in this case is a boy born at full term to a Caucasian mother and an African American father who are nonconsanguineous. The patient's mother did not receive full prenatal care. She underwent a limited ultrasound in the second trimester that documented only “active fetal movement and normal cardiac activity.” She was scheduled to have a complete anatomy scan, but the imaging was never completed. There was maternal drug use during the pregnancy, and the newborn's urine toxicology screen was positive for opioids and cocaine at birth. There was no documented limb bowing on newborn physical exam. He was placed in the care of a foster family for several months and then was returned to the care of his biologic parents. Family history was negative for short stature (mother's height: 162 cm and father's height: 195 cm), early loss of primary or secondary teeth, perinatal or early childhood deaths, or craniosynostosis.

**Figure 1:**
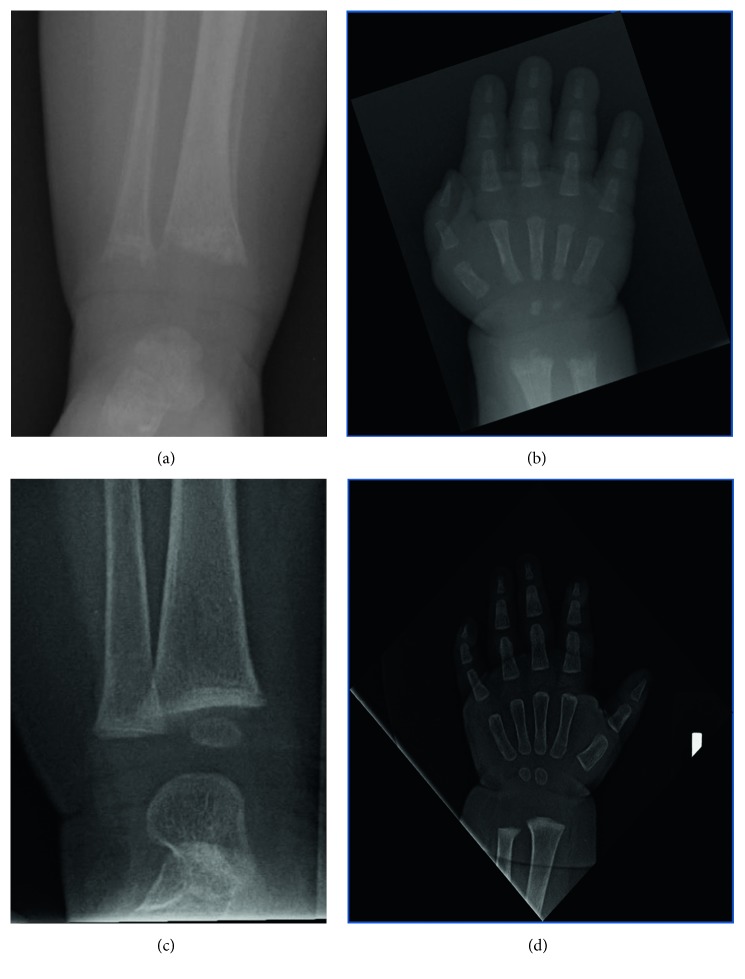
Initial skeletal survey performed at 8 weeks of age demonstrates fraying of the metaphyses of the long bones including the distal tibia (a) and radius and ulna (b). Incidentally note the benign periosteal reaction of the newborn about the tibia. Posttreatment images at 8 months of age (c, d). Only slight cupping of the metaphyses of the tibia (c) and radius and ulna (d) remains.

**Figure 2:**
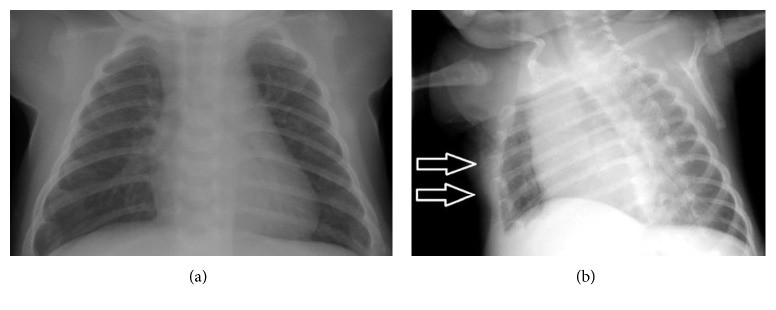
Pretreatment AP (a) and oblique (b) views of the chest show widening and cupping of the costochondral junctions.

At his 2-month-old well-child check, his primary care pediatrician's physical examination was notable for a hip click bilaterally. Given concerns for congenital hip dysplasia, his pediatrician attempted to order a hip ultrasound. Due to insurance issues, hip radiographs were alternatively obtained. Hip radiographs were not consistent with congenital hip dysplasia but were interpreted as demonstrating “abnormal appearance of the proximal femurs concerning for nonaccidental trauma,” and a follow-up complete skeletal survey was recommended. The skeletal survey was interpreted as demonstrating cupping and metaphyseal irregularities in the majority of the long bones and costochondral areas, most consistent with rickets (Figures 1 and 2). Baseline labs obtained by the pediatrician to evaluate for the underlying cause of rickets were notable for normal calcium, phosphorus, and parathyroid hormone. While his primary care pediatrician did not comment on it, his ALP was low at 33 units/L (reference range provided by the lab was 45–117 units/L and reference range for age and sex was 104–455 units/L) [13].

The patient was referred to pediatric endocrinology for further evaluation. Diffuse metaphyseal abnormalities on imaging, in addition to the patient's significantly low ALP, raised concern for HPP. Biochemical confirmatory testing for HPP included the urine phosphoethanolamine level elevated at 2228 nmol/mg creatinine (normal range: 0–372 nmol/mg creatinine, performed at Children's Hospital of Colorado) and serum vitamin B6 level elevated at 1030 mcg/L (normal range: 5–50 mcg/L, performed at Mayo Medical Laboratories New England). Genetic testing was performed by PreventionGenetics (Marshfield, Wisconsin). Two heterozygous, missense mutations in the TNSALP gene were identified that have previously been reported in patients with childhood or infantile HPP but have never been described together in one patient: c.1171C > T (p.Arg391Cys) and c.1077C > G (p.Ile359Met) [10, 14, 15].

The patient was also referred by his pediatrician to orthopedic surgery at 5 months of age. Physical exam was notable for minimal varus alignment of the lower extremities. Repeat hip radiographs were obtained at that time, which revealed interval worsening of rachitic changes in the ileum and proximal femoral metaphyses compared to previous imaging.

Growth parameters at birth were as follows: the birth weight was at the 15th percentile, the length was at the 43rd percentile, and the head circumference was at the 9th percentile. During the first 6 months of life, the patient's length dropped and remained between the 3rd and the 5th percentiles. The head circumference was also small, remaining at the 5th percentile. Weight gain, however, was appropriate and progressed to the 23rd percentile at 2 months and then to the 48th percentile at 5 months.

Given the patient's poor linear growth, diffuse metaphyseal changes, and worsening metaphyseal changes on repeat imaging, he was started on asfotase alfa 2 mg/kg three times weekly at 7 months of age. Repeat imaging obtained after 1 month of asfotase alfa, when the patient was 8 months old, revealed only slight cupping of the metaphyses of the tibia and radius and ulna (Figure 1). Repeat imaging obtained after 1 year of asfotase alfa, when the patient was 19 months old, revealed no appreciable abnormalities of the metaphyses.

The patient's growth parameters also improved during the course of treatment. His length improved from the 3rd to the 5th percentile in the first 6 months of life, to the 11th percentile after 4 months, and to the 25th percentile after 1 year of asfotase alfa. The head circumference progressed from the 5th percentile at 6 months of age to the 51st percentile after 4 months and the 66th percentile after 1 year of asfotase alfa. This patient otherwise demonstrated no gross motor delay and walked at the age of 13 months. He has never experienced any long bone fractures.

Prior to initiation of treatment, the patient had experienced eruption of only 1 tooth, which had not been spontaneously lost. In the first year on asfotase alfa, he experienced eruption of 14 teeth with subsequent loss of 4 teeth. The roots of these lost teeth were intact. The patient tolerated treatment with asfotase alfa well with no side effects, including no injection site reactions or hypersensitivity reactions.

## 3. Discussion

Here, we report a case of HPP that was diagnosed prior to 6 months of age after a workup was undertaken by his pediatrician for a suspicion of congenital hip dysplasia, followed by additional imaging that was obtained given concern for nonaccidental trauma on the part of the interpreting radiologist. The skeletal survey was interpreted at 2 months of age as being consistent with rickets, including metaphyseal cupping and fraying in multiple long bones. He also demonstrated poor linear growth and mild limb bowing. The diagnosis of HPP was confirmed biochemically (elevated serum pyridoxine phosphate and urine phosphoethanolamine) and with TNSALP mutation analysis (2 missense mutations identified). He overall demonstrated a mild but progressive course in the first 6 months of life with worsening metaphyseal changes noted on imaging between 2 months of life and 5 months of life. He was started on enzyme replacement therapy, after which he demonstrated improvement in radiologic bony abnormalities and linear growth. This case highlights several important challenges in the diagnosis, classification, and management of HPP.

HPP is a very rare condition, and it is optimistic to expect most primary care physicians to consider HPP in a differential diagnosis [1]. However, it is important to note that an infant with radiographic findings similar to rickets with low ALP should be a clue for the clinician to broaden the differential diagnosis [2]. Use of age- and gender-specific normative values becomes important for interpretation of ALP levels, as the normal range for ALP is higher in younger children [2, 13]. Frequently, diagnostic laboratories do not report age appropriate ranges, which may lead the clinician to overlook diagnoses such as HPP or to investigate age-normal ALP levels as elevated.

The diagnosis of HPP can be made when medical history, physical exam, laboratory studies, and radiographic findings are consistent [1]. Persistently low serum ALP activity using age- and gender-specific reference ranges is a hallmark of HPP [16]. Additional laboratory studies that support the diagnosis include elevated serum pyridoxal 5′-phosphate (vitamin B6) and elevated serum or urine phosphoethanolamine levels [1]. TNSALP mutation analysis can also be performed. While not indispensable to the diagnosis of HPP, TNSALP mutation analysis is essential for understanding inheritance patterns and recurrence risk in future pregnancies [1, 16].

In this case, mutational testing revealed that the patient is most likely a compound heterozygote with 2 heterozygous, missense mutations in the TNSALP gene. Given that mutational testing is not available in parents, it is also possible that this patient carries two mutations on the same allele. Both mutations have previously been reported in patients with childhood and infantile HPP, but to our knowledge, they have never previously been reported in the same individual [6, 10, 14, 15]. Unique to this patient is the ethnic background of his parents; his mother is a Caucasian, and his father is an African American. HPP is particularly rare in individuals of black ancestry. One review of 278 families with HPP revealed only 1 kindred with black ancestry [17]. Unfortunately, parents declined mutational analysis, and we could not confirm that his father is indeed a carrier.

An additional challenge is to determine the classification of HPP once the diagnosis is established because prognosis and management vary significantly among the types of HPP. HPP presents in an incredibly broad range of severities, spanning from death in utero to isolated dental complications [1]. There are five traditional classifications of HPP in addition to the more recently described entity of benign prenatal HPP, but even within classification groups, there exists a range of severities (Table 1) [1, 9].

The patient in this case presented with radiologic findings and mild clinical symptoms prior to 6 months of age. The first evidence of radiographic rickets was present on a hip radiograph and the skeletal survey obtained when the patient was 2 months old. He had an additional hip radiograph at 5 months of age that demonstrated worsening rachitic changes in the ileum and proximal metaphyses of the bilateral femurs. In the first 6 months of life, he demonstrated poor linear growth in addition to mild limb bowing that was first noted on physical examination at 5 months of age. While the early presentation of his findings would traditionally classify him as infantile HPP, he did not demonstrate other classic findings of infantile HPP such as failure to thrive, hypotonia, delayed motor milestones, or craniosynostosis in the first 6 months of life [1]. While the timeline of the development of radiologic findings, after the perinatal period but before 6 months of age, is consistent with infantile HPP, his relatively mild clinical manifestations are somewhat atypical of this more severe form of HPP.

Childhood HPP classically presents after 6 months of age but has a wide range of expressivity and can present with relatively mild symptoms [1, 6]. Mild childhood HPP features premature painless deciduous loss of teeth with intact roots and rickets, which may manifest only radiographically [6]. The patient in this case could theoretically represent a case of mild childhood HPP diagnosed early during evaluation for congenital hip dysplasia. In this case, the patient may have continued to have a relatively mild clinical course without initiation of enzyme replacement therapy.

Benign prenatal HPP is another consideration in classifying this patient's HPP. BP-HPP is characterized by abnormal prenatal ultrasound, including poor skeletal mineralization or limb bowing, or abnormal physical exam findings at birth, including limb bowing or skin dimpling, followed by a relatively mild postnatal clinical course [9, 18]. In the reported patient, no abnormalities were noted on his limited prenatal ultrasound, and he had no noted limb bowing or skin dimpling on newborn physical examination. Although his prenatal ultrasound was interpreted as normal, the exam was limited, and minor in utero skeletal changes could potentially have been considered a normal variant by the interpreting physician. Patients with BP-HPP often have a mild postnatal course; however, several published cases have demonstrated radiographic worsening on skeletal findings prior to improvement, including worsening bowing at 6 months of age and worsening osteopenia at 9 months of age [9]. Similarly, our patient demonstrated worsening rachitic changes in the ileum and proximal femoral metaphyses between 2 and 6 months of age. Although his prenatal imaging and newborn physical exam are not entirely consistent with BP-HPP, this patient's relatively mild postnatal course makes BP-HPP an important consideration.

When left untreated, the prognosis for infantile HPP is poor, with an estimated 50% mortality during infancy [11]. The cause of death in most cases is related to respiratory insufficiency, generally leading to ventilator dependence and other respiratory complications [10]. Enzyme replacement therapy, asfotase alfa, was approved for use in HPP in 2015 [1]. Asfotase alfa has been reported to be effective in the treatment of life-threatening perinatal and infantile forms of HPP [10, 11]. Outcomes include increased survival, less respiratory compromise, and improved radiographic evidence of skeletal mineralization [11]. Efficacy has also been shown in older children (6–12 years old) with infantile and childhood HPP, including significant healing of skeletal manifestations, better stature, and improved strength and agility [12].

We initiated asfotase alfa treatment because of the patient's widespread and progressive radiologic changes in addition to poor linear growth. There were demonstrable improvements in metaphyseal fraying and cupping within 1 month of initiating therapy, followed by almost complete resolution of all metaphyseal abnormalities after 1 year of therapy. His linear growth also improved from the 3rd percentile prior to treatment to the 25th percentile at 1 year on asfotase alfa. Of course, this improvement may be the natural evolution of the condition if this patient indeed has BP-HPP.

Finally, this patient experienced spontaneous loss of four deciduous teeth after the initiation of therapy. An early asfotase alfa study in perinatal and infantile hypophosphatasia found that only one patient had HPP-related loss of a tooth after initiation of therapy [10]. This finding in our patient suggests that asfotase alfa may not alter the natural history of premature deciduous tooth loss in some cases, perhaps depending upon the classification of HPP.

In summary, we report a case of HPP diagnosed early in life based on radiologic and biochemical findings. Genetic analysis revealed that the patient was compound heterozygous for two previously described missense mutations in the TNSALP gene. The classification of his HPP remains unclear. However, the patient demonstrated improvement in radiographic findings and linear growth after treatment with asfotase alfa. This case highlights the importance of correct interpretation of ALP levels using age- and gender-specific normal ranges, the wide clinical spectrum of HPP, and the challenges in its management.

## Figures and Tables

**Table 1:** Classification and clinical manifestations of HPP [1–3, 6, 8, 9].

Classification	Age at onset	Clinical manifestations
Perinatal	In utero or at birth	Most severe form; symptoms apparent at birth:
		(i) In utero skeletal changes (profound hypomineralization of the cartilage and bone)
		(ii) Bony abnormalities: short and deformed limbs and soft calvarium
		(iii) Respiratory compromise at birth to the first week of birth
		(iv) Pyridoxine-dependent seizures
		(v) Nearly always fatal soon after birth

Benign perinatal	In utero or at birth	(i) Skeletal deformities or hypomineralization in utero
		(ii) Limb bowing, skin dimples, and rickets as neonates
		(iii) Mild postnatal course with spontaneous improvement in bony symptoms (can range from odonto-HPP to infantile HPP)

Infantile	After birth, before 6 months of age	(i) Respiratory failure within weeks to months of birth
		(ii) Bony abnormalities: deformity of the thorax, fractures, and craniosynostosis
		(iii) Poor feeding and failure to thrive
		(iv) Delayed motor milestones
		(v) Proptosis and mild hypertelorism
		(vi) Pyridoxine-dependent seizures
		(vii) Hypercalcemia and hypercalciuria

Childhood: mild or severe	After 6 months, before 18 years of age	Wide range of severities:
		(i) Early loss of primary dentition
		(ii) Bony abnormalities: misshapen skull, tibial bowing, enlarged joints from metaphyseal flaring, and recurrent and poorly healing fractures
		(iii) Bone pain
		(iv) Gross and fine motor delay

Adult	After 18 years of age (usually middle age)	(i) Loss of adult dentition
		(ii) Bony abnormalities: recurrent fractures with poor healing and low bone mass
		(iii) Crystal arthropathy
		(iv) Muscle weakness

Odonto-HPP	Before 4–5 years of age	(i) Isolated early loss of primary dentition with intact tooth root
		(ii) No other characteristic radiologic or histopathologic manifestations
